# The Effect of Power Distance Belief on the Evaluation of Experiential and Material Purchases

**DOI:** 10.3390/bs13040314

**Published:** 2023-04-06

**Authors:** Hongsub Kim, Yeosun Yoon, Byoungsoo Kim

**Affiliations:** 1 KAIST College of Business, Seoul 02455, Republic of Korea; 2 School of Business, Yeungnam University, Gyeongsan 38541, Republic of Korea

**Keywords:** experiential purchases, material purchases, power distance belief, comparison motivation, need for structure

## Abstract

Depending on the level of power distance belief (PDB), individuals have different motivations to compare themselves with other people. This study suggests that the relationship between purchase type (material versus experiential) and purchase evaluation is moderated by PDB. Furthermore, the effect of purchase type and PDB on purchase evaluation is mediated through comparison motivation. To investigate the effect of PDB on the evaluation, we conducted two experiments by manipulating a 2 (purchase type: material vs. experiential purchase) × 2 (PDB: low vs. high) between-subjects design. In the case of experiential purchases, individuals with high PDB exhibit lower purchase evaluations than those with low PDB, as they are more inclined to compare these with other experiential goods (study 1). Conversely, under material purchases, the impact of PDB on purchase evaluation does not differ as material purchases already motivate individuals to compare other material goods (study 1). Additionally, individuals with high PDB are more motivated to compare purchases due to their greater need for structure (study 2). Our findings provide guidelines for the development of advertising strategy with social networking services and live-streaming commerce platforms.

## 1. Introduction

Imagine two friends, John and Mike, both of whom have recently returned from their vacations. While talking about their vacations, John evaluates his vacation by comparing it with Mike’s vacation. He compares his vacation with Mike’s as regards all the details, such as hotel service, the price of flight tickets, dinner had in local restaurants, among others. Comparing his vacation with Mike’s, John is sometimes very satisfied with his vacation but sometimes not. In contrast, Mike has less intention to compare his with John’s vacation. He thinks his vacation is unique, and it is hard to imagine what it would be like to go to John’s vacation instead of his.

As the preceding scenario illustrates, individuals have different evaluations about vacations depending on their information-processing tendencies. Because of these different information-processing tendencies, they have a different evaluation of their purchases of experience [1,2]. Although prior literature on experiential and material purchases has shown that purchasing an experience brings a greater hedonic value than purchasing a material good [3,4], personal traits would have an impact on how people evaluate their experiential and material purchases. Among various personal traits, a need for structure plays a vital role in influencing the evaluation of experiential and material purchase. A need for structure is defined as a personality trait that refers to an individual’s preference for order, organization, and predictability in their environment. Caprariello and Reis [5] found that individuals who are low in need for structure evaluated experiences more positively than material possessions.

Power distance belief (PDB) is defined as the extent to which individuals accept and endorse hierarchy and inequality in society. Several studies on PDB provide some evidence to understand the mechanism behind the evaluation of the purchase type [6,7]. Depending on the level of PDB, individuals have different motivations to compare themselves with other people [8]. Lee et al. [9] found that consumers high (vs. low) in PDB are less price-sensitive because they have a higher need for closure. In this research, we propose that PDB influences comparison motivation of the purchase, which, in turn, influences the hedonic value of the purchase. Specifically, this study suggests that, under the experiential purchase situation, individuals with high PDB are more likely to compare theirs with other experiential goods because of their elevated level of need for structure; thus, they are likely to have lower purchase evaluations. However, individuals with a low PDB have few motivations to compare other experiential goods; therefore, they are more likely to have higher purchase evaluations. Additionally, in the context of the material purchase, there is no difference in the purchase evaluation between customers with a high or low PDB because material purchases inherently motivate individuals to compare other material goods.

The goal of this research is an in-depth understanding of the role of a need for structure in the evaluation of material purchases and the evaluation of experiential purchases. This study investigated the moderating effect of PDB on the evaluation of experiential and material purchases. Our findings improve our understanding about the underlying mechanism that explains the relation between information-processing tendencies and the evaluation of purchases.

The findings of this article contribute to the existing literature in several ways. First, this study provides some evidences about the effect of PDB on purchase motivation by demonstrating that the effect influences the comparison motivation. Second, our present research contributes to the literature about material and experiential purchases by highlighting the role of PDB. Finally, this research also contributes to the literature on the need for structure by demonstrating it triggers a greater motivation to compare the purchase with other forgone options.

In the following section, we review relevant literature on the areas of experiential and material purchases and PDB. Next, we report two studies that investigate the effect of PDB and the purchase type on the evaluation of purchases. We conclude by discussing theoretical and practical contributions, implications and directions for further research.

## 2. Literature Review and Research Model

### 2.1. Experiential and Material Purchases

One classification of purchase type that received considerable attention over the last decades is experiential vs. material purchases [4]. The experiential purchase involves spending with “the primary intention of acquiring a life experience: an event or series of events that one lives through” and the material purchase involves spending with “the primary intention of acquiring a material good: a tangible object that is kept in one’s possession” [3]. For example, buying restaurant meals, concert tickets, theme park passes, or vacations are experiential purchases and buying furniture, clothing, smartphones, or televisions are material purchases.

Most of the work developed on experiential and material purchases to date has found that experience purchasing is typically more personally beneficial than material good-buying [3]. Compared to material buying, experiential buying leads to greater satisfaction [10], less regret of action [11], and greater happiness [4]. In addition, happiness from the experiential purchase is less adopted over time compared to the material purchase [12]. Importantly, a common finding from previous literature is that the benefits of acquiring an experiential purchase over a material purchase stem from the comparability of the purchase. For example, Carter and Gilovich [10] show that participants who are compensated with inferior material items rate their prizes less favorable, whereas there are no differences in the ratings of participants who receive experiential items as a prize. In addition, authors also suggest that salient comparisons have a greater impact on customer satisfaction with their material goods than with their experiential goods. Similarly, Rosenzweig and Gilovich [11] show that participants’ material purchase decisions are more likely to induce regrets of action. This regret would be affected by the tendency for experiences to be seen as more singular (i.e., less comparable) than material purchases.

Previous research also suggests why the hedonic value of the experiential purchase is less affected by the comparison [10]. Specifically, authors propose three reasons: (1) experiences are hard to compare prospectively (comparing two pamphlets of vacation vs. two wristwatches side by side); (2) experiences are hard to compare retrospectively (less regret of post-choice); and (3) individuals tend to examine unchosen material purchases more than unchosen experiential purchases.

Although prior research shows that the experiential purchase improves consumers’ well-being, the motivational factor influencing the perception of the purchase type is relatively uncovered. However, few systematic works investigate the effect of personal motivation on the perception of the purchase type [10,11]. Understanding the effect of personal motivation on the perception of the purchase type is important because it would help to examine the factors that influence personal evaluation.

### 2.2. Power Distance Belief, Comparison Motivation, and Evaluation of Purchases

PDB is defined as the degree to which individuals endorse hierarchy and accept inequalities in power [6,13]. Although there are inequalities in power within all societies, some are more accepting of hierarchy and legitimizing these inequalities than others [14,15]. The main difference between high and low PDB does not lie in the actual power disparity experienced by a person or the degree of power they possess, but in their attitudes towards power disparity [7,16]. Traditionally, PDB has been assessed at the country level (i.e., high in Malaysia, Mexico, and India, whereas low in Austria, Denmark, and Holland). However, recent research suggests that PDB is a psychological state that can also be studied by measuring it at the individual level, as well as through priming procedures [13,17]. Although power distance was the first cultural dimension identified by Hofstede [13], researchers have only recently begun to systematically examine its influence on consumer behavior.

A considerable amount of research into PDB has been investigating the effect on the information processing motivation of individuals. Importantly, some research has verified that cultures with differing levels of PDB have a different effect on the level of comparison motivation. For example, Guimond and his colleague [8] show that individuals in high (vs. low) power distance cultures display a more pronounced predisposition to social comparison. In their study, authors measure the PDB scale and the social comparison orientation scale of students from five countries (i.e., France, Belgium, the Netherlands, the United States, and Malaysia). The results show that there is a significant and positive relation between culture and social comparison orientation. For example, Malaysia, which has a high PDB culture, had the most pronounced predisposition to social comparison. Additionally, Kim and Zhang [18] suggest that individuals from high power distance cultures are sensitive to the difference in social status, and this status-seeking mindset makes consumers prefer brands that enhance their social status when compared to others. Cui et al. [19] report that individuals from high power distance cultures have higher purchase intentions for status goods than individuals from low power distance cultures in the context of a threat to self-worth. In summary, these lines of PDB research suggest that individuals with a high PDB have higher comparison motivation than individuals with a low PDB.

Thus, we propose that PDB tends to influence the comparison motivation of the purchase. In addition, PDB also affects the hedonic value of the purchase because it changes the comparison motivation. In other words, in the experiential purchase decision, customers with a high PDB are more likely to compare other experiential goods; thus, they are likely to have lower purchase evaluations. However, individuals with low PDB would have few motivations to compare other experiential goods; therefore, they are more likely to have higher purchase evaluations. Additionally, under the material purchase decision, the purchase evaluations between individuals with high or low PDB are almost the same because material buying already motivates customers to compare other material goods. Stated formally:

**H1a.**  *Under an experiential purchase situation, individuals with high power distance belief exhibit a lower purchase evaluation*.

**H1b.**  *Under a material purchase situation, the effect of power distance belief on the purchase evaluation does not differ*.

**H2.**  *The comparison motivation mediates the effect of power distance belief and the purchase type on the purchase evaluation*.

### 2.3. Power Distance Belief and a Need for Structure

Recent research has revealed that PDB is positively associated with a need for structure, which is a motivational variable representing a desire for clarity and order and an avoidance of ambiguity and gray areas [20]. Lalwani and Forcum [21] showed that individuals with a high PDB have a greater need for structure and tend to use price to judge quality. Consumers with a greater need for structure are more likely to be motivated to mentally discriminate among several brands, and to sort the brands [21]. The need for structure is regarded as the key antecedent that influences comparison motivation. Additionally, previous research on hierarchy suggests that PDB is connected to a need for structure. For example, Friesen et al. [22] found that hierarchies improve the utility of structure and order, compared to less hierarchical environments. In one study, participants were asked to rate a variety of words and phrases related to both hierarchy and equality, and the results indicated that hierarchy (versus equality) elicited greater structure, stability, coordination, predictability, and order. In another study, the authors found that participants with a greater preference for hierarchy have a higher need for structure.

Previous research has demonstrated that PDB affects the need for structure. However, to our knowledge, no prior research has examined the relationship between the need for structure and comparison motivation [22]. Nevertheless, research suggests that these variables are related [21]. Consumers with a greater need for structure are likely to be more motivated to mentally discriminate among brands and segregate them based on price because it is easy to compare prices between brands. Thus, we propose that individuals with high (vs. low) PDB are more likely to have comparison motivations regarding the purchase because of a greater need for structure. Stated formally:

**H3.**  *A need for structure mediates the effect of power distance belief and the purchase type on the comparison motivation*.

**Figure 1:**
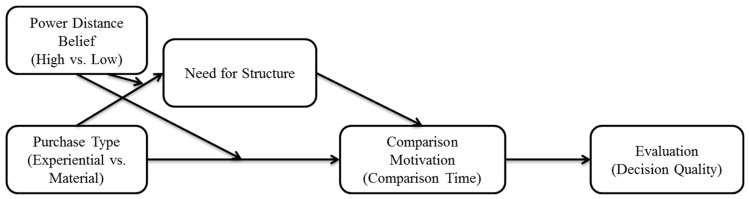
Research model.

Two studies are conducted to test the proposed theoretical model. Study 1 investigates the moderating role of PDB on the effect of the purchase type on purchase evaluation. Study 1 also provides the underlying process for the proposed interaction by examining the mediating role of comparison motivation. Study 2 extends the effect of PDB and the purchase type on comparison motivation by examining the mediating role of a need for structure. The theoretical model is presented in Figure 1.

## 3. Study 1

### 3.1. Participants and Procedure

Ninety-three participants (Mage = 37.4, 44 males) were recruited via Amazon Mechanical Turk (MTurk). They were randomly assigned to one of the conditions of a 2 (purchase type: material vs. experiential purchase) × 2 (PDB: low vs. high) between-subjects design. At the beginning of the survey, participants were told that they are going to complete two independent studies. We determined the minimum sample size for this study using the power analysis via G*Power 3.1.9.2 software [23]. We selected an effect size of 0.40, alpha of 0.05, and a power of 0.80, giving an acceptable sample size of 76 for this study. We checked repeated implausible answers or insincere responses, and all participants had no problem in their data quality.

First, they completed the priming task for PDB; participants’ level of PDB was primed using a sentence unscrambling task adapted from Zhang et al. [17]. This method allows for greater control over the experimental manipulation and can be easily adapted for use in a variety of cultural contexts [24]. Zhang et al. [17] used a sentence-unscrambling task to prime participants’ level of PDB and found that consumers with a higher level of PDB were more likely to make impulsive buying decisions. Self-construal at the country level can provide valuable insights into how cultural values influence consumer behavior and decision-making. Our participants were given 10 sets of scrambled words and for each set, they were asked to construct a complete sentence. Those in the high PDB condition were given 10 sets of scrambled words that are related to social hierarchy (e.g., “necessary, subordinates, to, superiors, our, social, order, obedience, from, is, for”) whereas those in the low PDF condition were given sets of words that are related to equality in a society (e.g., “not, necessary, subordinates, to, superiors, our, social, order, obedience, from, is, for”).

Following this, participants completed the manipulation task for purchase type as the second study. To manipulate the type of purchase, we constructed two different choice sets: one consists of 12 different material possessions (electronic products) and the other including 12 different options of experiences (vacations) [10]. Participants were given one of the two choice sets and asked to imagine themselves in the shoes of a person who is trying to make the best choice from the choice set. Each choice set consisted of three subcategories of products, each of which included four different choice options with varying attributes. For the material purchase condition, there were four flat-screen televisions, four smartphones, and four digital cameras included in the choice set. Similarly, for the experiential purchase condition, there were four beach vacations, four city vacations, and four national park vacations included as the choice options. Each choice option was described using two qualitative and two quantitative details. For example, one of the flat-screen television option was described with attributes such as “ultra HD performance/enhanced contrast (qualitative)” and “product price/consumer ratings from Consumer Reports (quantitative)”. Similarly, one of the beach vacation option was described using attributes including “short walk to bars, restaurants/various activities for nightlife (qualitative)” and “product price/consumer ratings from TripAdvisor.com (quantitative)”. To ensure that the participants felt that the choice options are realistic, the prices (ranging from USD 500 to USD 1999) and the consumer ratings (ranging from 3.20 to 4.15) were set to be positively correlated. The price levels and the consumer ratings across purchase type conditions were set to be similar.

Participants in each purchase type condition first saw all 12 options in the choice set for 5 s each. For each choice option, a picture, the product name, and the descriptions on four attributes were provided. They were then shown one of the choice options again and told to imagine themselves as a person who had chosen that option from the choice set they have seen previously. Following this, participants were shown a list of thumbnail images for all 12 choice options and told that they can look through the 12 choice options again by clicking on any of the thumbnail images. They were allowed to see the information on each option as much as they wanted. After participants finished reexamining the options, they responded to questions on their assessment of decision quality as well as the measures for manipulation check and alternative explanation variables. Finally, basic demographic information was collected and the participants were debriefed and thanked.

### 3.2. Measurement

#### 3.2.1. Participants’ Evaluation Measure

As our main dependent variable, we measured participants’ evaluation on the quality of the decision that is provided. We asked the participants to answer the following two questions adapted from a previous study [25]: “Given the options that were available, how good was this purchase decision?” (1 = very poor; 7 = very good), “To what extent do you think that this purchase decision was the very best choice from the options that were available?” (1 = worst choice possible; 7 = best choice possible). Two items were averaged to form an index of decision quality (r = 0.66, *p* < 0.001).

#### 3.2.2. Comparison Time Measure

To investigate the underlying mechanism of the effect of PDB and purchase type on decision quality evaluation, we measured the amount of time participants spent exploring the options as the comparison time (in seconds). The time spent to look through options other than the chosen option was collectively measured as the comparison time. Specifically, both the time spent looking through other options in the same subcategory and the time spent looking at the different subcategories were included in our measure. The time spent looking at the target purchase (the chosen option) was excluded from the measurement.

#### 3.2.3. Manipulation Checks and Other Measures

PDB priming was assessed using three items that were developed and validated by Zhang et al. [17]. They were provided with three sentences with blanks in the end including “For the time being, I am mainly thinking that                  .”, “At this moment, I feel that                 .”, and “On top of my mind right now are thoughts in agreement with saying                 .” For each of the sentences, they were given a 7-point bipolar scale where 1 represented “social equality is important” and 7 represented “social hierarchy is important”, and asked to indicate which expression seems appropriate for the blank in the sentence. The items were averaged to form a composite score of PDB priming (α = 0.88). A higher score indicated a high PDB whereas a lower score indicated a low PDB. 

The purchase type manipulation was assessed with one item from a previous study [10]. We asked participants to indicate their thoughts on the question “When you were thinking about and evaluating the chosen purchase, what was your thought about the chosen purchase?” on a 7-point bipolar scale where 1 represented “An Experience Which I Purchase to DO” and 7 represented “A Material Possession Which I Purchase to HAVE”. A higher score indicated that the participants were thinking about material purchases for possession whereas a lower score indicated that the participants were thinking about experiential purchases that are intended for action and experience.

To test and rule out potential alternative explanations, we asked the participants for their thoughts on the following two questions: “How much did the quality of other choice options influence your evaluation?” and “How much did the price of other choice options influence your evaluation?” (1 = not at all; 7 = very much). Furthermore, we also measured the participants’ dispositional self-construal level using a 12-item scale developed and validated by Oysermann [26].

### 3.3. Results

#### 3.3.1. Manipulation Checks

An independent-samples *t*-test was conducted to check for the PDB manipulation. The result showed that participants in the high PDB scored high in the PDB priming measure (M_high PDB_ = 4.99, M_low PDB_ = 4.24; t(91) = 2.762, *p* < 0.01). Additionally, another independent-samples *t*-test was conducted to check for the purchase type manipulation. The result showed that participants in the material purchase condition scored high in the purchase type check measure (M_material purchase_ = 5.00, M_experiential purchase_ = 2.02; t(91) = 10.797, *p* < 0.001). The results indicated both manipulations worked as intended. Gender, age, quality concern, price concern, and prior similar purchases were insignificant when induced as covariates (ps > 0.11).

#### 3.3.2. Participants’ Evaluation

**Figure 2:**
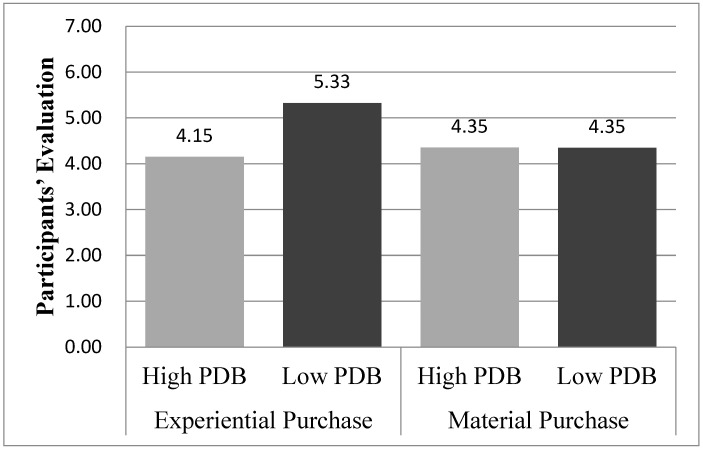
Result of study 1.

An ANOVA on the participants’ evaluation revealed a significant main effect of PDB (M_high PDB_ = 4.26, M_low PDB_ = 4.84; F(1,92) = 7.706, *p* = 0.007) and marginally significant main effect of the purchase type (M_material purchase_ = 4.35, M_experiential purchase_ = 4.74; F(1,92) = 3.407, *p* = 0.07). More importantly, the interaction effect of PDB and the purchase type on the participants’ evaluation was significant (F(1,92) = 7.875, *p* < 0.01). Follow-up planned contrasts supported the hypothesized results. As hypothesized, in the experiential purchase condition, participants with low PDB evaluate their hypothetical purchase higher than with high PDB (M_high PDB_ = 4.15, SD = 1.13 vs. M_low PDB_ = 5.33, SD = 0.90; F(1,89) = 15.418, *p* < 0.001). However, in the material purchase condition, there was no difference in the decision quality (M_high PDB_ = 4.35, SD = 1.12 vs. M_low PDB_ = 4.35, SD = 0.87; F(1,89) < 1, *p* = 0.98). These findings were consistent with hypotheses 1a and 1b (Figure 2).

#### 3.3.3. Mediating Role of the Comparison Time

To examine the extent to which comparison time mediated the effect of purchase type and PDB on decision quality, we tested a moderated mediation model using the bootstrapping procedure outlined by Preacher et al. [27]. Specifically, we utilized model 8 of the PROCESS macro developed by Hayes [28]. This approach involves procedures that calculate a 95% confidence interval (CI) around the indirect effect, i.e., the interactive effect of purchase type and PDB on decision quality via comparison time. The results revealed that the CI of the indirect effect of the highest-order interaction in the entire sample did not include zero (CI: 0.001 to 0.462; β = 0.126, SE = 0.130), which indicated mediation. In line with the predictions, in the experiential purchase condition, the CI for the indirect effect of PDB on the decision quality via the comparison time ranged from 0.016 to 0.298 (β = 0.110, SE = 0.077), which suggested that the comparison time differed according to the interaction of PDB and the purchase type differed in the decision quality. However, in the material purchase condition, the CI ranged from −0.210 to 0.069, which suggested that there was no difference in the comparison time and the decision quality. These findings were consistent with hypothesis 2.

#### 3.3.4. Alternative Explanations

Previous research shows that PDB has been associated with self-construal at the country level [13]. Although there is considerable research which shows that the effect of PDB is independent from those of self-construal [17,18,19], we considered it prudent to empirically ascertain the effect of self-construal on the purchase type. Thus, we conducted some analyses to rule out self-construal as a rival explanation. First, we conducted ANOVA on self-construal with PDB, the purchase type, and their interactions as independent variables. The results revealed that all effects were nonsignificant (all F(1,92) < 1, ps > 0.45). More importantly, the PDB manipulation did not affect self-construal measures (M_high PDB_ = 3.50, M_low PDB_ = 3.53; t(91) = −0.192, *p* > 0.84), suggesting that PDB and self-construal acted independently of each other. Additionally, another ANCOVA revealed that the interaction between PDB and the purchase type remained significant (F(1,92) = 8.164, *p* < 0.01) when self-construal was included in the model as a covariate; thus, our effects persisted after controlling for self-construal. To further assess the role of self-construal, we conducted a GLM on the decision quality with self-construal, the purchase type, and their interaction, which was similar to the previous ANOVA except for turning PDB to self-construal. The results revealed that the effect of self-construal and the interaction of self-construal and the purchase type on the decision quality were nonsignificant (all F(1,92) < 1, ps > 0.59). These findings revealed that the effect of PDB was different from those of self-construal.

### 3.4. Discussion

Study 1 examines the effect of PDB and the purchase type on the individuals’ evaluation of purchases. The results of study 1 are consistent with our hypotheses that under the experiential purchase situation, individuals with low (vs. high) PDB are more likely to have higher (vs. lower) purchase evaluations, whereas under the material purchase situation, there is no difference in the purchase evaluation between individuals with high or low PDB. Furthermore, we show that all of these effects on the evaluation of purchases are mediated by individuals’ comparison motivation. Additionally, the results of study 1 show that the difference in the evaluation of purchases is not due to the effect of self-construal but due to the effect of PDB, which is additional evidence that the effect of PDB is unique compared to those of self-construal.

Study 1 shows that the interaction of PDB and the purchase type affect the evaluation of purchases. In study 2, we measure the need for structure to examine the mediating role of structured thinking.

## 4. Study 2

### 4.1. Participants and Procedure

One hundred participants (M_age_ = 35.6, 48 males) were recruited via MTurk. All participants were native English speakers currently located in the United States. They were randomly assigned to one of the conditions of a 2 (purchase type: material vs. experiential purchase) × 2 (PDB: low vs. high) between-subjects design. Similarly to study 1, participants were first informed that they would complete several unrelated studies that were combined for the sake of efficiency. First, they completed the PDB priming task which was an opinion-writing task from Zhang et al. [17]. Their task was to read a statement about inequality and write a short essay either supporting or rejecting the statement including three supporting reasons. The presented statement was as follows: “There should be an order of inequality in this world in which everyone has a rightful place; high and low are protected by this order”, which was adopted from Hofstede’s [13] definition of PDB. Participants in the high (vs. low) PDB condition were asked to list three reasons to support (argue against) this statement.

Following this, participants were asked to complete the manipulation task for purchase type, which was identical to the one used in study 1. Participants were randomly assigned to the material or the experiential purchase condition and were presented a set of 12 choice options. As in study 1, they were told to imagine themselves as a person who has just made a choice among the 12 options and were given a chance to reexamine all options by clicking on a thumbnail image of each options. Afterwards, participants were asked questions measuring the participants’ need for structure as the process measures. They also answered questions for manipulation checks and alternative explanations. Finally, basic demographic information was collected and the participants were debriefed and thanked.

### 4.2. Measurement

#### 4.2.1. Comparison Time Measure

We measured the amount of time participants spent exploring the options as the comparison time in the same way we did in study 1. The comparison time included the total time spent reexamining the choice options that are not chosen. The time spends looking at the target purchase was excluded.

#### 4.2.2. Need for Structure Measure

The need for structure was measured using the 10-item scale validated by Webster and Kruglanski [29]. A sample item included “I enjoy having a clear and structured mode of life”. (1 = strongly disagree; 7 = strongly agree). The responses were averaged to form an index of the need for structure.

#### 4.2.3. Manipulation Checks and Other Measures

The effectiveness of the PDB priming task was assessed using the same manipulation check items used in study 1. The responses to the three items were averaged to form a composite score of PDB priming (α = 0.92). A higher score indicated high PDB, whereas a lower score indicated that the participants had situationally low PDB. Similarly, the purchase type manipulation was also assessed using the same item as in study 1. A higher score indicated that the participants were thinking about material purchases for possession, whereas a lower score indicated that the participants were thinking about experiential purchases that are intended for action and experience. Finally, the same alternative explanation variables from study 1 were also measured to test and rule out alternative explanations.

### 4.3. Results

#### 4.3.1. Manipulation Checks

An independent-samples *t*-test was conducted to check for PDB manipulation. The results showed that participants in the high PDB scored high in PDB priming measure (M_high PDB_ = 5.60, M_low PDB_ = 4.60; t(98) = 3.983, *p* < 0.001). Additionally, another independent-samples *t*-test was conducted to check the purchase type manipulation. The result showed that participants in the material purchase condition scored high in the purchase type check measure (M_material purchase_ = 5.36, M_experiential purchase_ = 2.12; t(98) = 11.927, *p* < 0.001). The results indicated that both manipulations worked as intended. Gender, age, quality concern, price concern, and prior similar purchases were insignificant when induced as covariates (ps > 0.17).

#### 4.3.2. Comparison Time

**Figure 3:**
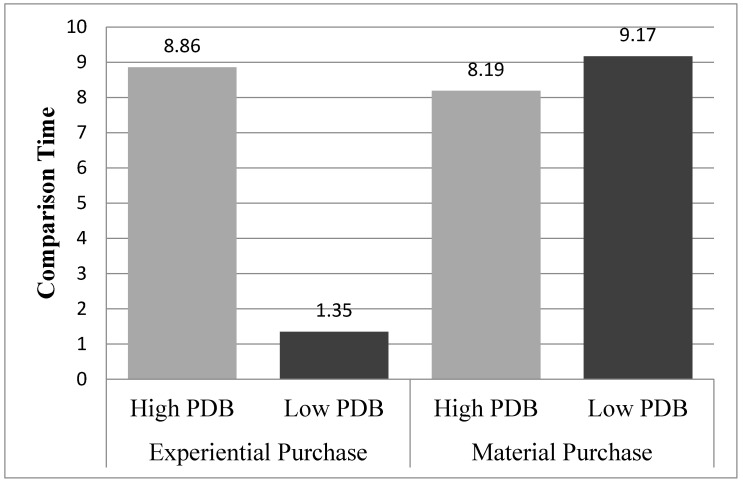
Results of study 2.

An ANOVA conducted on the decision quality revealed a marginally significant main effect of the purchase type (M_material purchase_ = 8.98, M_experiential purchase_ = 4.95; F(1,99) = 3.167, *p* = 0.08) but nonsignificant main effect of PDB (M_high PDB_ = 8.53, M_low PDB_ = 5.53; F(1,99) = 1.927, *p* = 0.17). More importantly, the interaction effect of PDB and the purchase type on decision quality was significant (F(1,99) = 4.378, *p* = 0.04). Follow-up planned contrasts revealed that, in the experiential purchase condition, participants with low PDB spent less time exploring other options than those with high PDB (M_high PDB_ = 8.86, SD = 13.03 vs. M_low PDB_ = 1.35, SD = 2.79; F(1,96) = 6.057, *p* = 0.02). However, in the material purchase condition, there was no difference in the comparison time (M_high PDB_ = 8.19, SD = 11.13 vs. M_low PDB_ = 9.17, SD = 13.01; F(1,96) < 1, *p* = 0.62). Figure 3 shows the results of study 2.

#### 4.3.3. Mediating Role of Need for Structure

To examine the extent to which the need for structure mediated the effect of the purchase type and PDB on the comparison, we tested the moderated mediation model using the bootstrapping procedure described by Preacher et al. [27]. As in study 1, we used model 8 of the PROCESS macro by Hayes [28]. This approach includes procedures that compute a 95% confidence interval (CI) around the indirect effect (i.e., the interactive effect of the purchase type and PDB on the comparison time via the need for structure). The results revealed that the CI of the indirect effect of the highest-order interaction in the entire sample did not include zero (CI: 0.432 to 3.426; β = 1.481, SE = 0.713), which indicated mediation. In line with predictions, in the experiential purchase condition, the CI for the indirect effect of PDB on the comparison time via a need for structure ranged from 0.617 to 3.459 (β = 1.723, SE = 0.703), which suggested that a need for structure differs according to the interaction of PDB, and the purchase type differed in the comparison time. However, in the material purchase condition, the CI ranged from −0.498 to 1.615, which suggested that there was no difference in the need for structure and in the comparison time. These findings were consistent with hypothesis 3.

### 4.4. Discussion

Study 2 examines the effect of PDB and the purchase type on the comparison time, and more importantly, highlights the mediating role of the need for structure as a function of PDB and the purchase type. As predicted, under the experiential purchase situation, individuals with low (vs. high) PDB are more likely to compare fewer (vs. more) options, whereas, under the material purchase situation, there is no difference in comparison time between individuals with high or low PDB. Additionally, also consistent with our hypotheses, we show that all of these effects on the comparison time are mediated by a need for structure.

## 5. General Discussion

### 5.1. Theoretical and Practical Implications

The objective of the current research was to examine the interaction effect of PDB and the type of purchase on the product evaluation, as well as the underlying mechanisms. Through a series of studies, we show that under the experiential purchase situation, individuals with low (vs. high) PDB are more likely to have higher purchase evaluations, whereas under the material purchase situation, there is no difference in the purchase evaluation between individuals with high or low PDB. Specifically, in study 1, we demonstrate that the interaction effect of PDB and the purchase type on the product evaluation is mediated through comparison motivation. Subsequently, in study 2, we also show that the interaction of PDB and the purchase type affects the need for structure, which influences comparison motivation. Additionally, in study 1, we showed that the effect of PDB on product evaluation is independent of self-construal.

Our findings offer contributions to the literature on PDB, the purchase type, and the need for structure. First, we enriched the emerging literature on the effect of PDB on purchase motivation by demonstrating that the effect influences to comparison motivation. In doing so, we extended previous research about PDB, which has revealed an effect on status consumptions [7,18] or impulsive buying [17]. Specifically, we showed that individuals with high (vs. low) PDB are more likely to have comparison motivation regarding their purchase due to a greater need for structure, which advances the PDB literature.

Second, the present research contributes to the literature about material and experiential purchases by highlighting the role of PDB. Although most studies on purchase type focus on the characteristics of purchases which consequently affect consumer well-being [30], recent research shows some factors that influence the perception of the purchase type [31]. Our research adds to this body of literature by demonstrating that PDB influences comparison motivation and the purchase evaluation.

Lastly, we also contribute to the literature on the need for structure by demonstrating that it triggers greater motivation to compare the purchase with other forgone options. Although previous research only suggests the relation between a need for structure and comparison motivation [21], we shed light on the causal relationships between these variables by showing the tendency of individuals with a higher (vs. lower) need for structure to compare with more options.

Our findings provide important practical implications. The present study can provide guidelines for the development of advertising strategies with social networking services and live-streaming commerce platforms. Recently, a significant number of small companies have advertised their products in social networking services and live-streaming services because it is cost-effective and makes it easy to show the user experiences with a short movie clip. However, when communicating on these platforms, marketers should exercise caution. For example, if they are struggling with marketing promotion which emphasizes experiences with their products, they can selectively promote their products in countries with low PDB culture or advertise a message that emphasizes equality and egalitarianism. This way, managers can improve their product evaluation, further leading to higher brand evaluations.

### 5.2. Limitations and Future Research Directions

Nevertheless, it is important to consider several limitations. Firstly, the sample of this study is confined to participants from a single country, thus limiting its generalizability. Future research could expand the diversity of the sample to enhance the study’s external validity. Additionally, a cross-cultural comparison could be meaningful in testing the proposed model. Second, this study investigated whether a need for structure mediates the effect of power distance belief and the purchase type on the comparison motivation. Although a need for structure is an important personal trait, future research should analyze whether other personal traits, such as extraversion, agreeableness, and openness, impact comparison motivation and evaluation [32]. Finally, in future research, it is necessary to re-test the findings of this study based on sufficient samples in order to generalize our results.

## Data Availability

The data presented in this study are available on request from the corresponding author.
